# Associations between perceived and observational physical environmental factors and the use of walking paths: a cross-sectional study

**DOI:** 10.1186/1471-2458-14-627

**Published:** 2014-06-20

**Authors:** Ying-nan Jia, Hua Fu

**Affiliations:** 1 School of Public Health, Key Lab of Public Health Safety of the Ministry of Education, Fudan University, Shanghai 200032, China; 2 Department of Preventive Medicine, School of Public Health, Fudan University, Dong’an Road 130, Shanghai, China

**Keywords:** Walking path utilization, Direct observation, Perceived and observed environment, Chinese residents

## Abstract

**Background:**

How to promote physical activity is an important public health problem that is attracting increasing attention. Although the application of environmental approaches is believed to promote resident walking, there remains insufficient evidence of the effectiveness of these interventions.

**Methods:**

This study employed direct observation and questionnaires. Observations were performed on each Tuesday, Thursday, Saturday, and Sunday from April 13th to May 16th. Fourteen trained observers observed six community walking paths, and an additional walking path in a park. The trained observers filled out 2388 observation forms in the field, including 228 forms rating the permanent environment, and 1080 forms assessing the current environment and counting the number of walkers. A total of 1800 questionnaireswere administered to community residents.

**Results:**

The results of both observation and questionnaires showed good association regarding the characteristics of walking path users (for observation, female = 54.4%; for questionnaire interviews, female, OR = 1.441), and the environmental features associated with walking path utilization (for observation, positive associations were observed between the utilization index and observational environmental variables; for questionnaire interviews, roads and aesthetics were important, OR = 1.044). There were positive associations between path use and time, a preference for brisk walking, and the observed current and permanent environmental variables. Female participants were more likely to use walking paths than males (OR = 1.441, 95% confidence interval [CI] 1.126–1.846). BMI and traffic hazard safety were significantly negatively associated with walking path use (OR = 0.948, 95% CI 0.915–0.981, and OR = 0.933, 95% CI 0.887–0.981, respectively). Roads, aesthetics, and knowledge of physical activity were significantly positively correlated with use of walking paths (OR = 1.044, 95% CI 1.017–1.072, and OR = 1.175, 95% CI 1.043–1.323). Participants that resided further than 1 km from the park were less likely to use walking paths (OR = 0.703, 95% CI 0.530–0.933). Gender-specific associations were also found.

**Conclusions:**

Both perceived and objective environmental factors were associated with walking path use. Data suggested that the permanent and current conditions of the paths might influence walking path utilization, and that gender-specific promotion strategies should be considered.

## Background

Regular physical activity is an important contributor to health that reduces the risk of many chronic diseases such as hypertension and diabetes [1-3]. With changes in modern working and lifestyles, the number of people who participate in moderate and vigorous physical activity during their leisure time is generally <20%, and physical activity levels decline in Chinese adults, particularly in the middle-aged working population [4-6]. The increasing prevalence of physical inactivity in China is placing significant burdens on public health.

How to promote physical activity is an important public health problem that is attracting increasing attention. Previous studies assessing the determinants of physical activity with the aim of developing interventions have focused on individual demographics and psychosocial factors [7]. More recently, studies revealed either positive or no associations between the built environment and physical activity [8-14]. The application of environmental approaches to promote physical activity has received attention in many developed countries. Healthy People (2010) identified objectives focused on improving access to physical activity facilities, such as walking and cycling trails [15]. Because there are significant differences between the living conditions, population density, and work demands between developed countries and China due to economic and cultural factors, it is likely that the residents’ cognition, environmental needs, and physical activity behavior would also differ. For example, in urban China the per capita living space is 31.6 m^2^, with a population density of 2209 people/km^2^. The population density in Shanghai is even greater, with >3630 people/km^2^. Because walking is the most common recreational physical activity in the Chinese population [16], the Chinese government have built or improved walking paths in many neighborhoods in Shanghai. Compared with the construction of other physical activity facilities, it is most feasible to build or improve walking paths that are based on original paths in communities because of the high population density and centralized residences [17]. In addition, these environmental interventions are believed to have a more long-term impact than other interventions, such as providing pedometers [18].

Although building or improving walking paths can provide access for most residents, the effectiveness of these interventions remains unclear. Previously, walking path utilization was investigated using questionnaires, and the walking path environments were assessed by geographic information system (GIS) or questionnaires [19,20]. However, these two approaches have some limitations. First, the environmental assessment of walking paths performed previously was not comprehensive and precise. Specifically, GIS and scales could assess the objective and perceived environment of the fixed area, but not the physical features of each walking path [21,22]. Therefore, previous studies failed to evaluate the permanent physical features and current conditions of the walking paths, which might have a direct impact on walking path utilization. Second, walking path use is likely to change with environmental variations, such as lighting conditions and cleanness. Time-specific associations between the environment and walking path utilization were not assessed adequately in previous studies. Therefore, it is necessary to assess the environment and walking path utilization simultaneously.

This study is the first to combine the use of questionnaires with direct observation to resolve the limitations of assessing walking path utilization, and to define potential environmental correlates. Direct observation allowed the path utilization and environment to be assessed in separate time periods. Moreover, it allowed the environment to be assessed precisely, including the permanent physical and current conditions of each walking path. The results of this study provide novel empirical evidence demonstrating the relationship between environmental factors and physical activity behavior.

## Methods

### Subjects and participants

#### Observation of walking path utilization

We had paid a visit to 10 communities and sport parks before performing the study. An intercept convenient sample of 20 community residents (>20 years old) from several neighborhoods was interviewed. The results revealed that length of the walking path and the surroundings might influence walking path utilization. Therefore, six walking paths in the neighborhoods were selected according to the permanent physical and current conditions of each path. An additional walking path in a park was also observed to provide reference information. The walking path in sport park was also in Minhang district as same as the other six paths. However, it was located in the park rather than a neighborhood. The park was built for recreation and exercises for all the residents in the district. A description of the seven walking paths is presented in Table 1.

**Table 1:** Description of the walking paths

**Walking path**	**Length (m)**	**Area covered (1000 m**^**2**^**)**	**Population (thousands)**	**Location**
A1	144	6.78	4.128	Border
A2	134	5.00	0.919	Border
B1	210	9.00	1.830	Center
B2	210	1.55	0.750	Center
C1	338	7.90	1.619	Corner
C2	313	2.67	2.590	Gate
C3*	300	/	/	Park

*Unlike the other walking paths, C3 was located in the park. As the area covered by C3 and population were difficult to estimate, the utilization index cannot be computed.

Fourteen trained observers observed seven walking paths in the community and park. The trained observers filled out 2388 observation forms in the field, including 228 forms rating the permanent environment, and 1080 forms assessing the current environment and counting the number of walkers.

#### Questionnaire interviews

The subjects were selected during two-stages of sampling. During the first stage, 17 different neighborhoods were selected randomly in the Minhang district of Shanghai, China. Among all the 17 neighborhoods, there were six neighborhoods where the paths were observed. The average distance from park to their neighborhoods was 4.15 km. In the second selection stage, 1800 participants aged 15–75 years were identified randomly after stratifying by gender and age. A total of 1528 valid questionnaires were returned, which was a response rate of 84.9%. Trained interviewers were responsible for collecting the self-administered questionnaires during door-to-door visits, as long as the respondents were able to understand the questionnaires. For the respondents who were unable to understand the questionnaires, interviewers were responsible for explaining the questions. Those questionnaires with invalid or missing data (n = 272) were excluded from the data analyses.

### Procedures and the development of study protocols

#### Observation of walking path utilization

Direct observation was used to assess the use of walking paths in the community based on the different features of the paths and their surroundings [23]. Direct observation was performed on each Tuesday, Thursday, Saturday, and Sunday between April 13th and May 16th. Four periods of observations were used: the morning (6:30–7:30 AM), noon (11:30–1:30 PM), afternoon (3:30–4:30 PM), and evening (6:30–7:30 PM). The observation periods consisted of 45 min observation with 15 min rest intervals were designed. Because the park closed early, observation in Sport Park, located in the center of the district, was implemented for only three periods a day, without the evening. Fourteen trained observers recorded information describing the environmental characteristics of seven walking paths. Each observer took 15 minutes to examine and rate the permanent and current environment of a pre-determined walking path before the start of each observation period. The number of walkers who passed the target area was defined as times. If the same person passed the target area more than once, then additional times were recorded. During the observation periods, observers counted the times, and recorded the age, gender, and walking speed of the walkers. Because the length of the walking path might affect its use, seven walking paths in the community were divided into three groups (groups A, B, and C) according to their length. A new indicator, the utilization index, was calculating using times, observing units, and the population of the community to assess the impact of the population on path use (utilization Index = times/observing unit/covering population × 1000).

A rating scale for the environmental features of the walking paths, including current and permanent assessments, was developed based on literature reviews and intercept interviews with local residents [24-27]. Two observers were assigned to each path at the same time, but rated the parameters independently. The mean of the two ratings was then reported as the result of each observational interval.

#### Assessment of the environmental features of walking paths

Observers rated the walking paths based on two sets of scale: the permanent, and current rating scales. The same tables were applied to assess the permanent environment and current environment for all the paths. All the observers were required to rate the permanent environment of all the walking paths once. During each observation period, the two observers need to rate the current environment of paths and record the times. Permanent physical features included the path length, aesthetics, path material, resting areas, path slope, the placement of healthy signs along the paths, and the prohibition of vehicles. These seven items were weighted with different scores based on the perception of 20 residents regarding the contribution of the environment to physical activity. The maximum score for aesthetics was 25, those of length, material, and healthy signs were 15, and that for the remaining three items was 10. The current rating scale was developed based on lighting, cleanness, and accessibility conditions, and the organization of walking activities. The maximum score of organization of walking activities was 10, and that for other three items was 30.

### Questionnaire interviews

#### Demographic variables

Self-reported demographic variables included gender, age, education, employment status, height, bodyweight, family income, and marital status.

#### Assessment of physical activity

The self-reported data collected from the Chinese version long International Physical Activity Questionnaire (IPAQ) were summarized within each physical activity parameter to estimate the total time spent in occupational-, transport-, household-, and leisure-related physical activity, as well as the total time spent sitting each week. The IPAQ was shown to be reliable and valid [28,29]. The IPAQ survey and scoring protocols (available online at: http://www.ipaq.ki.se) were adopted for this study. There were three levels of physical activity used to classify populations: low, moderate, and high. The two criteria for classification as “high” were (a) vigorous intensity activity on at least 3 days, achieving a minimum total physical activity of at least 1500 metabolism equivalent (MET)-minutes/week or (b) 7 or more days of any combination of walking, moderate-intensity, or vigorous-intensity activities, achieving a minimum total physical activity of at least 3000 MET-minutes/week. “Moderate” activity was classified as: (a) 3 or more days of vigorous-intensity activity of at least 20 minutes per day, or (b) five or more days of moderate-intensity activity and/or walking for at least 30 minutes per day, or (c) five or more days of any combination of walking, moderate-intensity, or vigorous intensity activities achieving a minimum total physical activity of at least 600 MET-minutes/week. Individuals who did not meet the criteria for “moderate” or “high” were considered to have a “low” level of physical activity.

#### Assessment of environment walkability and knowledge of physical activity

Because of the environmental and cultural differences between western countries and China, a revised Abbreviated Neighborhood Environment Walkability Scale for Chinese residents (CNEWS) was developed. We pre-determined three communities with good, modest, and poorly walkable environments based on prior observations. Twenty adults were then selected from these three communities. Each individual was interviewed face-to-face using the CNEWS. All the residents were asked to sort the variables based on their perception of the contribution of the environment to physical activity. A score would then be given to each item according to the sorting of the residents. Three items (trees, sanitation, and crime safety) were rated as the most important, and were weighted with double scores. An additional convenient sample of 360 residents who lived in the community (>20 years old) from two neighborhoods with different walkable environments were interviewed by intercept investigators. Data revealed that the CNEWS for urban community residents had good reliability and validity, and thus could be used as an effective tool for assessing the walkable environment in urban communities in China [22].

A neighborhood-level walkability index with four parameters (accessibility to services, neighborhood surroundings/aesthetics, traffic safety, and crime safety) was calculated using the CNEWS, which had shown good reliability (intra-class range 0.802 - 0.947) [22]. Access to services includedfour items (stores, parks, bus/train stops, and other places). Neighborhood surroundings/aesthetics included seven items (trees, sanitation, lighting, condition of the road, natural sights, interesting things, and places for walking). Traffic safety included three items (traffic accidents, obstruction of traffic, and speed of traffic). Crime safety included three items (total crime rate, crime rate during the day, and crime rate at night). The score of each item was ranged from 1 to 5: 1 = strongly disagree, 2 = somewhat disagree, 3 = unsure, 4 = somewhat agree, and 5 = strongly agree. Therefore, a higher value indicated a more physical activity-friendly environment.

In addition to the perceived environmental variables, the distances from the participants’ communities to the park were also measured using professional instruments on an electronic map. Subjects were then separated in to two groups based on distances less than or greater than 1 km. Subjects’ knowledge of physical activity behavior was assessed in three questions regarding the benefits and recommendations of regular physical activity in adults.

### Ethical permission

The study received approval from the ethics committee of the School of public health of Fudan University, China 51 (IRB00002408&FWA00002399). Written informed consent statements were obtained from all participants. The right to withdraw and autonomy of the respondents were also explained.

### Analyses

For direct observational data, descriptive and correlation analyses were employed to explore the potential factors that influence the use of walking paths. For data from the questionnaire interviews, analysis of variance and chi-squared tests were performed to compare the differences between two groups that used or did not use the walking paths. Multiple logistic regression analysis was employed to identify potential factors that influenced the use of walking paths. The independent variables consisted of four perceived neighborhood environmental variables: access to services, roads and aesthetics, traffic safety, and crime safety. BMI, knowledge of physical activity, distance to the park, and daily sedentary time were also analyzed.

Apart from distance to the park, the other variables were assessed as continuous variables. Odds ratios were adjusted by gender, age, education, employment status, marital status, and family income. Gender-specific multiple logistic regression analyses were also performed to explore the differences between males and females. For all analyses, statistical significance was set at 0.05. Statistical analysis was performed using the Statistical Package for Social Sciences (SPSS IBM 20.0).

## Results

### Demographics of the walking paths users by direct observation

Table 2 shows the demographic characteristics of the walking paths users, as assessed by direct observation. The path users were mostly middle-aged and elderly, accounting for 58.8 and 33% of the users, respectively. There were also slightly more female than male users. The walking paths were used mostly in the morning (41.6%).

**Table 2:** Characteristics of the walking paths users determined by direct observation

		**Times**	**%**			**Times**	**%**
Age (yr.)	≤19	1359	8.2	Time	Weekday	7789	46.9
	20 - 59	9771.5	58.8		Weekend	8825.5	53.1
	≥60	5484	33.0		Total	16614.5	100.0
	Total	16614.5	100.0		Morning	6915.5	41.6
Walking path	A1	2193.5	13.2		Noon	2238.5	13.5
	A2	319.5	1.9		Afternoon	4312	26.0
	B1	1147	6.9		Evening	3148.5	18.9
	B2	378.5	2.3		Total	16614.5	100.0
	C1	493.5	3.0	Gender	Male	7584	45.6
	C2	5330.5	32.1		Female	9030.5	54.4
	C3	6752	40.6		Total	16614.5	100.0
	Total	16614.5	100.0				

The utilization of the path labeled C2 was extremely high because the path was placed at the only entrance to the neighborhood, and many residents had to pass it for daily commuting; these individuals were counted as walkers during direct observation. Unlike the other paths, the use of path C2 was even throughout the four observation times across the day. C3 is a walking path that was built especially for leisure walking in the local park, providing the best conditions of all seven paths. Residents from a large area could come to use it, and it exhibited the highest times of usage.

The consistency of observational variables was calculated in Table 3. The consistency ranged from 72.7% to 94.7%, which showed an acceptable reliability

**Table 3:** The consistency of observational variables

**Variables**		**Observation period**	**Consistency (%)**		
			**Method of calculation**	**Means**	**SD**
Score	Current rating	540	A/B	92.6	16.9
	Total times	540	A/B	94.7	12.4
Gender	Male	540	A/B	92.6	16.9
	Female	540	A/B	93.0	16.4
Speed	Times of brisk walking	540	A/B	72.8	33.6
	Times of casual walking	540	A/B	72.7	31.7
Age	≤19	540	A/B	90.2	23.4
	20-59	540	A/B	84.4	23.7
	≥60	540	A/B	74.3	34.5

(A and B were the results from a pair of observers at same time and same place).

Table 4 showed the association between walking path use and observational environmental variables, including lighting conditions, accessibility, cleanness, and organization of the activities. Times were significantly positively correlated with all four observational environmental variables. Brisk walking is recommended as a more effective form of exercise compared with casual walking by local health authorities. Brisk walking can improve one’s cardiovascular fitness more effectively than casual walking. Therefore, the prevalence of brisk walking was also calculated, and was considered to be an index measuring the walking quality. The prevalence of brisk walking was significantly positively associated only with path accessibility and cleanness.

**Table 4:** Associations between walking path use and observational environmental variables

**Variable**	**Observing units**	**Times**		**Brisk walking prevalence**	
		***r***	***P***	***r***	***P***
Lighting condition	1080	0.101	<0.05	0.000	>0.05
Accessibility	1080	0.180	<0.05	0.061	<0.05
Cleanliness	1080	0.130	<0.05	0.146	<0.05
Organization of activities	1080	0.076	<0.05	0.006	>0.05

As shown in Table 5, path location was the main factor that influenced the utilization index. When walking paths were classified by location, A1 and A2 were in pair 1, and B1 and B2 were in pair 2. Positive associations between the utilization index and observational environmental variables, including permanent and current ratings, were represented in both pairs 1 and 2. Similar associations between the prevalence of brisk walking and observational environmental variables were also found in each group.

**Table 5:** Environmental assessment rating of the walking paths by observational methods

**Walking path**	Utilization Index*	**Brisk walking prevalence (%)**	**Permanent rating**	**Current rating**	**Overall rating**
A1	3.25	53.30	69.53 ± 6.01	85.09 ± 8.86	77.31
A2	2.17	35.89	40.29 ± 5.21	70.59 ± 14.62	55.44
B1	3.89	37.01	79.47 ± 3.61	84.19 ± 10.64	81.83
B2	3.15	31.86	64.71 ± 4.60	70.44 ± 14.19	67.58
C1	1.91	38.74	60.91 ± 8.08	77.25 ± 12.93	69.08
C2	12.86	50.98	58.13 ± 3.97	79.50 ± 12.62	68.82
C3	/	76.99	94.97 ± 6.13	87.58 ± 6.95	91.28

*Utilization Index = Times/Observing Unit/covering population*1000.

### Participant characteristics and representation

The demographics of the study participants are shown in Table 6. The final sample consisted of 1,528 participants (49.2% male, 50.8% female) aged 15–75. Of the participants, 33.9% were overweight (body mass index (BMI) ≥24 kg/m^2^, the national criteria for being overweight in mainland China), 74.6% had been married, 32.7% had a college/university degree, and 45.3% were employed. The main demographic characteristics of walking path users were female gender and middle age.

**Table 6:** Subject characteristics of the investigated community residents

**Variable**	**All respondents**	**Walking path users**	**Non-users**	***χ***^**2 **^**(*P*)**
	**n (%)**	**n (%)**	**n (%)**	
Gender (%)				10.935 (0.001)
Male	752 (49.2)	195 (42.8)	557 (52.0)	
Female	776 (50.8)	261 (57.2)	514 (48.0)	
Age				
15-19 years	104 (6.8)	24 (5.3)	80 (7.5)	28.667 (0.000)
20 - 59 years	1072 (70.2)	287 (62.9)	784 (73.2)	
60 - 75 years	352 (23.0)	145 (31.8)	207 (19.3)	
Marital status				
Unmarried/Divorced/Widowed	388 (25.4)	88 (19.3)	300 (28.0)	12.811 (0.000)
Married	1140 (74.6)	368 (80.7)	771 (72.0)	
Education				
Junior high school	568 (37.2)	190 (41.7)	378 (35.3)	10.728 (0.013)
High School/technical secondary school	457 (29.9)	136 (29.8)	321 (30.0)	
Junior college	208 (13.6)	63 (13.8)	144 (13.4)	
Bachelors/Masters/Doctorate	295 (19.3)	67 (14.7)	228 (21.3)	
Employment status				
Employed	692 (45.3)	178 (39.0)	514 (48.0)	38.495 (0.000)
Student	164 (10.7)	35 (7.7)	129 (12.0)	
Retired	445 (29.1)	181 (39.7)	264 (24.6)	
Unemployed	115 (7.5)	36 (7.9)	79 (7.4)	
Others	112 (7.3)	26 (5.7)	85 (7.9)	
Family income (Missing = 110)				
<2000RMB	748 (52.8)	226 (53.6)	522 (52.5)	0.897 (0.639)
2000 - 3999RMB	481 (33.9)	136 (32.2)	344 (34.6)	
>4000RMB	189 (13.3)	60 (14.2)	129 (13.0)	
BMI (Missing = 31)				
≤18.5	139 (9.3)	44 (9.8)	95 (9.1)	5.766 (0.056)
18.5 - 24	841 (56.2)	271 (60.2)	570 (54.5)	
≥24	517 (34.5)	135 (30.0)	381 (36.4)	
Distance to sport park (C3)				
≤ 1 km	367 (24.0)	138 (30.3)	229 (21.4)	13.817 (0.000)
>1 km	1161 (76.0)	318 (69.7)	842 (78.6)	
Leisure-time physical activity				
Low	888 (58.1)	200 (43.9)	688 (64.2)	57.357 (0.000)
Moderate	492 (32.2)	189 (41.4)	303 (28.3)	
High	148 (9.7)	67 (14.7)	80 (7.5)	
	Mean ± SD			F (P)
Knowledge of physical activity	1.39 ± 1.07	1.48 ± 1.07	1.35 ± 1.06	4.753 (0.029)
Daily sedentary time(min)	703.58 ± 256.68	682.05 ± 263.52	713.35 ± 252.62	4.784 (0.029)
Access to services	14.22 ± 2.95	14.39 ± 3.00	14.15 ± 2.92	2.169 (0.141)
Roads and aesthetics	30.01 ± 5.60	30.99 ± 5.73	29.59 ± 5.49	20.270 (0.000)
Traffic safety	10.66 ± 2.54	10.57 ± 2.54	10.69 ± 2.54	0.749 (0.387)
Crime safety	13.87 ± 3.12	14.05 ± 3.27	13.80 ± 3.05	2.023 (0.155)

Compared with non-users, walking path users were more highly represented by female, senior, and married residents. The distribution of family income was similar between the walking path users and non-users. However, the distribution of other variables including employment status, BMI, and education was different between users and nonusers. A significantly higher proportion of users reported a distance to the park (C3) of <1 km compared with non-users. Path users were more represented by participants who reported moderate and high levels of leisure-time physical activity, less sedentary time, and had more knowledge of physical activity. Of the four perceived environmental variables, walking path users perceived better roads and aesthetics than non-users; these observations were significant.

### Results of logistic regression analysis

Results of logistic regression are shown in Table 7. Female participants were more likely to use the walking paths than males (OR = 1.441, 95% CI 1.126–1.846). BMI and traffic hazard safety score were significantly negatively associated with the use of the walking paths (OR = 0.948, 95% CI 0.915–0.981, and OR = 0.933, 95% CI 0.887–0.981, respectively). Roads, aesthetics score, and knowledge of physical activity were significantly positively correlated with walking path use (OR = 1.044, 95% CI 1.017–1.072, OR = 1.175, 95% CI 1.043–1.323). Participants who reported a distance to the park (C3) of >1 km were less likely to use the walking paths (OR = 0.703, 95% CI 0.530–0.933).

**Table 7:** Multivariate analysis - logistic regression of variables for walking path use between male and female participants

	**Total (n = 1388)**		**Male (n = 680)**		**Female (n = 708)**	
	**OR (95% CI)**	**P value**	**OR (95% CI)**	**P value**	**OR (95% CI)**	**P value**
Gender		0.004				
Male (ref)	1.00					
Female	1.441 (1.126-1.846)					
Age		0.122		0.676		0.157
15 - 29 years (ref)	1.00		1.00		1.00	
30 - 59 years	0.720 (0.350-1.481)	0.371	0.871 (0.301-2.521)	0.799	0.672 (0.242-1.865)	
60 - 75 years	1.028 (0.462-2.288)	0.947	1.170 (0.338-4.057)	0.804	1.025 (0.339-3.096)	
Marital status		0.258		0.550		0.359
Unmarried/Divorced/Widowed (ref)	1.00		1.00		1.00	
Married	1.236 (0.856-1.784)	0.258	1.181 (0.685-2.037)	0.550	1.269 (0.762-2.114)	0.359
Education		0.192		0.095		0.202
Junior high school (ref)	1.00		1.00		1.00	
High School/technical secondary school	1.008 (0.747-1.360)	0.958	0.634 (0.396-1.016)	0.058	1.383 (0.926-2.065)	0.113
Junior college	1.115 (0.754-1.648)	0.586	0.912 (0.518-1.606)	0.750	1.227 (0.706-2.131)	0.469
Bachelors/Masters/Doctorate	0.697 (0.458-1.061)	0.092	0.517 (0.278-0.960)	0.037	0.822 (0.457-1.479)	0.514
Employment		0.291		0.751		0.522
Employed (ref)	1.00		1.00		1.00	
Student	0.930 (0.494-1.754)	0.824	0.886 (0.344-2.282	0.802	0.957 (0.398-2.302)	0.922
Retired	1.507 (1.033-2.197)	0.033	1.570 (0.788-3.129)	0.200	1.507 (0.945-2.403)	0.085
Unemployed	1.118 (0.686-1.822)	0.655	1.071 (0.436-2.632)	0.881	1.211 (0.667-2.199)	0.528
Others	1.251 (0.734-2.131)	0.411	1.254 (0.545-2.884)	0.595	1.303 (0.639-2.657)	0.467
Family income		0.057		0.015		0.807
<2000RMB (ref)	1.00		1.00		1.00	
2000 - 3999RMB	1.097 (0.833-1.444)	0.510	1.126 (0.738-1.718)	0.581	1.041 (0.720-1.506)	0.829
>4000RMB	1.628 (1.091-2.427)	0.017	2.391 (1.313-4.355)	0.004	1.203 (0.691-2.096)	0.513
BMI	0.948 (0.915-0.981)	0.002	0.926 (0.880-0.975)	0.003	0.968(0.921-1.016)	0.190
Distance to sport park		0.015		0.070		0.177
≤1 km (ref)	1.00		1.00		1.00	
>1 km	0.703 (0.530-0.933)	0.015	0.669 (0.433-1.034)	0.070	0.770 (0.526-1.125)	0.177
Knowledge of physical activity	1.175 (1.043-1.323)	0.008	1.155 (0.958-1.393)	0.131	1.179 (1.007-1.381)	0.040
Daily sedentary time (min)	1.000 (0.999-1.000)	0.060	1.000 (0.999-1.000)	0.332	1.000 (0.999-1.000)	0.123
Accessibility to services	1.000 (0.957-1.044)	0.988	0.980 (0.914-1.050)	0.568	1.007 (0.952-1.066)	0.799
Roads and Aesthetics	1.044 (1.017-1.072)	0.001	1.078 (1.036-1.123)	0.000	1.015 (0.980-1.052)	0.402
Traffic safety	0.933 (0.887-0.981)	0.007	0.901 (0.838-0.969)	0.005	0.962 (0.895-1.033)	0.283
Crime safety	1.018 (0.973-1.065)	0.447	0.989 (0.922-1.060)	0.747	1.045 (0.982-1.112)	0.168

**p < 0.005, *p < 0.05.

When analyses were stratified by gender, data revealed gender-specific associations between potential correlates and walking path use. In males, BMI and the traffic hazard safety score were significantly negatively associated with walking path use (OR = 0.926, 95% CI 0.880–0.975, and OR = 0.901, 95% CI 0.838–0.969, respectively). In contrast, the roads and aesthetics scores were positively correlated with walking path use significantly (OR = 1.078, 95% CI 1.036–1.123). Participants who reported family income >4000 RMB were more likely to use walking paths than those who reported an income <2000 RMB (OR = 2.391, 95% CI 1.313–4.355). Among females, knowledge of physical activity was the only variable that was significantly associated with walking path use (OR = 1.179, 95% CI 1.007–1.381).

## Discussion

Very few studies are available describing the leisure-time walking behavior of community residents in China, particularly those that assess factors that influence walking path use. In this study, direct observation and questionnaires were employed to collect data to assess these parameters. Specifically, we described the characteristics of residents who used the walking path for walking as a health-enhancing physical activity, the features of the walking paths that influenced residents’ decisions to use them, and residents’ preferred walking path features.

In general, the results of observation and investigation showed a good association for identifying the characteristics of walking path users (for observation: female, 54.4%; for questionnaire interviews: female, OR = 1.441), and environmental features that were associated with walking path use (for observation: positive associations were observed between the utilization index and observational environmental variables; for questionnaire interviews, roads and aesthetics were important, OR = 1.044). These two methods supported and complemented each other, each determining demographic characteristics of walking path users and identifying potential factors that influenced path use. Results from the questionnaire focused on whether perceived environmental factors were associated with path use. Although logistic regression analyses examined the association between potential influencing factors and path utilization, they failed to explore whether the frequency of walking path use and the quality of walking were associated with the environmental features of the paths. Therefore, direct observation was a necessary complement to the questionnaire, and provided a relatively objective assessment of the permanent and current environmental features of each oath. In addition, times and utilization indices were employed to assess the frequency of walking path use. A prevalence of brisk walking was also considered to assess the quality of walking path use.

Results from observation revealed that the path users were mostly middle-aged and elderly, and that there were fewer male than female users. These observations are consistent with a previous study [19]. Univariate analysis revealed increased path use among married, low educated, and retired individuals. This differs from a previous study, which demonstrated that participants with more education and higher income were more likely to use walking paths [19]. These inconsistent findings could be explained by the possibility that residents with a higher education and income level were more likely to live in better neighborhood surroundings, but were more physically inactive due to busy social and work schedules [6]. According to the results of logistic regression analysis, walking path users were more likely to be physically active in their leisure time. Groups of participants who were more likely to have used the walking paths included those who reported distance to the park (C3) of <1 km, those with less sedentary time, more knowledge of physical activity, and those with better perceived better roads and aesthetics. Consistent with our findings, a previous study of walking paths also reported that distance to the walking paths was negatively correlated with their use [20].

In addition to the characteristics of walking path users, another primary focus of this study was to examine the association between both perceived and objective environmental factors and walking path use. The results of direct observation revealed positive associations between time, brisk walking prevalence, and observational current environmental variables including lighting conditions, accessibility, cleanness, and the organization of activities. Although the correlations between time and observational environment were low, they might actually be good in the field of environment and PA because there were many potential influencing factors of PA, such as gender, age, education. As shown in Table 5, location is likely to be the main factor that influences the utilization index. After classifying the walking paths by location, positive associations between utilization index and observational environmental variables (including permanent and current ratings) were represented in both pair 1 and 2. Similar associations between a brisk walking prevalence and observational environmental variables were also found. Some studies performed in developed countries reported results that were consistent with our study [30,31]. These data suggest that not only permanent, but also current conditions affect path use.

In the multiple logistic regression models, a combination of sociodemographic and environmental variables, including aesthetics and traffic safety, showed statistically significant associations with walking path use. Consistent with a previous study that showed positive associations between park access and walking [32], access to the Sport Park was also positively correlated with use of the walking paths. It is noteworthy that traffic safety was negatively associated with path use. Because most walking paths were located in neighborhoods, people who perceived poor traffic safety were more likely to walk inside the neighborhood rather than use the sidewalks outside. Although some previous studies reported associations between crime safety and physical activity, and gender-specific associations in developing countries such as Nigeria and Brazil [33,34], perceived crime safety was not associated with walking path use in our study. Gender-specific associations between crime safety and walking were also not identified. However, these results were consistent with Duncan’s meta-analysis [35]. Because Shanghai is generally perceived to be safe, differences in the perception of crime safety might be insufficient to demonstrate any potential associations.

Recently, an increasing number of studies have focused on the gender-specific associations between the environment and walking, and have reported varying results [36,37]. Because gender was the only significant demographic variable identified in our study, gender differences in the association between the perceived environment and walking path use were examined. In males, BMI, traffic safety, and roads and aesthetics were significantly associated with walking path use. Participants who reported a family income >4000 RMB were more likely to use walking paths than those with a family income <2000 RMB. These environmental correlated findings were partially consistent with previous studies [37,38]. In females, knowledge of physical activity was the factor that influenced walking path use significantly. Other studies also found that knowledge of physical activity was correlated with increased physical activity [39]. A possible explanation for this gender-specific observation is that females were more likely to listen to health-related advice than males. Compared with females, males might need more support from the physical environment, such as good traffic safety and aesthetics. These findings suggest that different strategies should be considered to promote the use of walking paths, such as health education for females and improving the environment for males.

There are some limitations in this study. First, the direction of causality could not be addressed due to the cross-sectional study design. Second, some subjective bias existed since 20 observers reported the results of the observations. Moreover, although the rating scale for the environmental features of the walking paths was developed based on literature reviews and intercept interviews with local residents, the reliability and validity of the scale have not been tested. In spite of these limitations, this study provides novel evidence on walking and the use of walking paths in China, and suggests that walking paths might be helpful to promote leisure-time activity in China.

## Conclusion

There was consistency regarding the demographic characteristics of walking path users and potential influencing factors determined using the observation and questionnaire interviews. Results suggested that both perceived and objective environmental factors were associated with the use of walking paths. In addition, the permanent and current conditions of the paths might influence walking path utilization, and gender-specific promotion strategies should be considered.

## Abbreviations

GIS: Geographic information system; IPAQ: International physical activity questionnaire; CNEWS: Neighbourhood environment walkability scale for Chinese; SPSS: Statistical package for social sciences; BMI: Body mass index.

## Competing interests

The authors declare that they have no competing interests.

## Authors’ contributions

YJ participated in the design of the study, performed the statistical analysis, and drafted the manuscript. YJ also performed data acquisition and interpretation. HF conceived the study, and participated in its design and coordination. HF was involved in drafting the manuscript or revising it critically for important intellectual content. Both authors read and approved the final manuscript.

## Pre-publication history

The pre-publication history for this paper can be accessed here:

http://www.biomedcentral.com/1471-2458/14/627/prepub
